# *Porphyromonas gingivalis* secreted factors drive epithelial–mesenchymal transition (EMT) through gingipains and an H_**2**_S-mediated bacterial defense system

**DOI:** 10.1080/19490976.2026.2647532

**Published:** 2026-03-24

**Authors:** Michal Kazelnik, Rana Masri, Michal Caspi, Amnon Wittenstein, Ilan Tsarfaty, Elhanan Borenstein, Tal Caller, Konstantin Shatalin, Evgeny Nudler, Rina Rosin-Arbesfeld

**Affiliations:** a Department of Clinical Microbiology and Immunology, Gray Faculty of Medical and Health Sciences, Tel Aviv University, Tel Aviv, Israel; b Clinical Microbiology and Immunology, Blavatnik School of Computer Science, Tel Aviv University, Tel Aviv, Israel; c Neufeld and Tamman Cardiovascular Research Institutes, Gray Faculty of Medical and Health Sciences, Tel Aviv University, Tel Aviv, Israel; d Sagol Center for Regenerative Medicine, Gray Faculty of Medical and Health Sciences, Tel Aviv University, Tel Aviv, Israel; e Biochemistry & Molecular Pharmacology, New York University Grossman School of Medicine, New York, NY, USA; f Howard Hughes Medical Institute, New York, NY, USA

**Keywords:** *Porphyromonas gingivalis*, colorectal cancer, epithelial–mesenchymal transition (EMT)

## Abstract

Dysbiosis of the gut microbiota is strongly associated with a wide range of pathologies, including various types of cancer. Porphyromonas gingivalis *(P. gingivalis*), an oral bacterium, is implicated in the development of colorectal cancer (CRC), and although the exact mechanisms by which *P. gingivalis* contributes to CRC remain unclear, and emerging evidence suggests that various bacterial elements are involved in the bacterium's pathogenic effects. Here, we show that *P. gingivalis* secreted factors promote CRC neoplasia progression by modulating both the Wnt/β-catenin and the Hippo–YAP signaling pathways. Using specific inhibitors and *P. gingivalis* mutant strains, our findings demonstrate that cysteine proteases, specifically Lysin-gingipain (*Kgp*) and Argin-gingipain A (*RgpA*), as well as hydrogen sulfide (H₂S), strongly induce the expression of epithelial–mesenchymal transition (EMT) markers, leading to cell detachment and increased motility. These findings reveal a novel connection between microbial virulence and defense mechanisms, such as H₂S, and host cell transformation, suggesting a potential role for bacterial secreted factors in driving CRC neoplasia.

## Introduction

Colorectal cancer (CRC) is one of the most common and deadliest cancer types.^1^^,^^2^ The development of CRC is a multistep complex process in which combinations of genetic mutations and epigenetic changes induce malignant transformation. One major cause for the initiation of numerous CRC cases is hyperactivation of the canonical Wnt signaling pathway.^3^ Oncogenic Wnt signaling overactivation can be initiated by different mechanisms, including mutations in components of the Wnt cascade, which lead to the nuclear accumulation of β-catenin and induced gene transcription,^4^ as well as by epigenetic modifications. Alongside stimulating cellular proliferation, Wnt signaling affects a broad spectrum of cancerous characteristics in the human colon, including apoptosis, inflammation, and epithelial‒mesenchymal transition (EMT),^5^ which is a hallmark of cancer cell movement and invasion. Other core cellular signaling pathways, such as the Hippo pathway, are also involved in the development of CRC.^6^ The epigenetic changes in colon carcinogenesis include the interaction between cancer cells and the tumor microenvironment (TME), where immune responses play a significant role.^7^ An important factor that affects the colonic TME is the gut microbiome,^8^^,^^9^though oral microbiota dysbiosis is also strongly associated with gastrointestinal disorders.^10-14^ Specifically, in CRC tumorigenicity,^15-19^ the gram-negative oral bacterium *Fusobacterium nucleatum* (*F. nucleatum*)^20^^,^^21^ and *Porphyromonas gingivalis* (*P. gingivalis*)^22^ are implicated. *F. nucleatum* is the best-characterized species associated with CRC and is frequently detected in clinical samples from CRC patients, where it has been shown to contribute to tumor initiation, progression, and immune modulation. Though less studied, increasing evidence also connects *P. gingivalis* to colorectal carcinogenesis. While most studies examining the association between *P. gingivalis* and CRC have relied on *in vitro* systems and murine models, a recent clinical study investigated the detection of *P. gingivalis* in fecal and mucosal samples from CRC patients, where its presence correlates with poorer cancer-specific survival.^23^ Experimental studies using CRC cell lines and animal models have reported similar tumor-promoting effects, providing mechanistic support for a potential role of *P. gingivalis* in colorectal carcinogenesis. The pathogenic action of *P. gingivalis* is facilitated by numerous secreted and cellular virulence and defense factors. The secreted factors contain the gingipains proteases, which account for 85% of the total proteolytic activity of the bacteria. Other bacterial virulence factors include fimbriae, hemolysin, hemagglutinins, outer membrane vesicles, and lipopolysaccharides (LPS), as well as the short-chain fatty acid (SCFA) butyrate. In addition to virulence factors, *P. gingivalis* possesses defense mechanisms that counteract the host response, and some of these factors, such as gingipains, serve dual roles in both virulence and defense. One of the defense systems shared by many anaerobic bacteria is the production and secretion of hydrogen sulfide (H_2_S), which protects bacteria from oxidative stress, antibiotics, and host immunity.^24^ H_2_S is generated by the bacterial cystathionine γ-lyase (CSE), cystathionine β-synthase (CBS), and 3-mercapto pyruvate sulfurtransferase (3-MST) enzymes.^24^

The suggested mechanisms involved in the CRC-mediated pathogenicity of *P. gingivalis* include bacterial adherence to colonic epithelia,^25^ activation of the MAPK/ERK signaling pathway,^26^ induced expression of the deubiquitinase UCHL3,^27^ and enhancement of inflammation by affecting tumor-infiltrating myeloid^22^ and invariant natural killer T (iNKT) cells.^28^ Importantly, it has been demonstrated that bacterial secretion of the short-chain fatty acid butyrate, typically associated with an anti-inflammatory response, induces cellular senescence and pro-inflammatory gene expression, and is partially involved in CRC tumorigenesis.^29^ Here, we aimed to define and explore other secreted products of *P. gingivalis* that may contribute to CRC progression.

## Results

### *P. gingivalis* cell-free supernatant (CFS) induces canonical Wnt signaling

**Figure 1.**
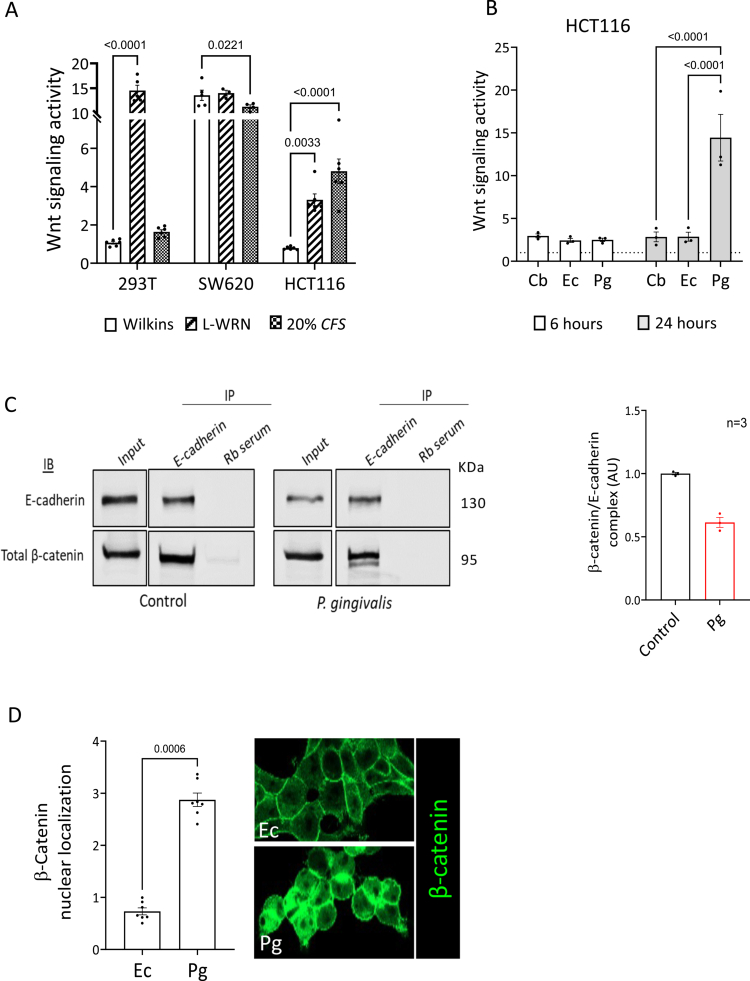
The cell free supernatant (CFS) of *P. gingivalis* induces canonical Wnt signaling. (A) The HEK293T, SW620, and HCT116 cell lines were transfected with the TOPFLASH system vectors for 48 h and subjected to luciferase assay following incubation with 20% *Pg-*CFS or LWRN CM (medium containing the Wnt ligand). A two-way ANOVA with Holm‒Šídák's multiple comparisons test was employed; *p-*values are indicated in the graphs. (B) HCT116 cells were treated with 20% CFS from *C. butyricum*, *E. coli,* and *P. gingivalis* bacteria for 6 h or 24 h. The cells were harvested and subjected to the TOPFLASH/FOPFLASH Luciferase assay. The results were normalized to the Wilkins broth control. A two-way ANOVA with Holm‒Šídák's multiple comparisons test was employed; *p-*values are indicated in the graphs. (C) HCT116 cells were incubated with *Pg*-CFS for 24 h. Protein extracts were immunoprecipitated with E-cadherin antibody or rabbit serum as a control, followed by western blot (WB) analysis using total β-catenin and E-cadherin antibodies (Left). Band intensity was quantified using ImageJ software and normalized by dividing the intensity of β-catenin with that of E-cadherin, and the control (right). The non-parametric Mann–Whitney test was used. (D) HCT116 cells were incubated with 20% *Pg*-CFS or 40% *E. coli*-CFS for 24 h, then stained with an anti-β-catenin antibody and visualized using confocal microscopy. An independent script was used to quantify the RGB intensity of nuclear β-catenin in 7 fields (3 independent repeats) of each sample. A non-parametric Mann‒Whitney test was employed; the *p*-value is indicated on the graph. Pg = *P. gingivalis*, Ec = *E. coli*, and Cb = *C. butyricum.*

As overactivation of the canonical Wnt cascade is a hallmark of CRC development, we tested the effect of *P. gingivalis* CFS (henceforth: *“Pg*-CFS”) on Wnt signaling activity. HEK293T naïve cells and SW620 and HCT116 CRC cells were treated with *Pg*-CFS, L-WRN-conditioned medium (which contains Wnt3a, R-spondin 3, and Noggin, which activates the Wnt pathway), or control medium. The HEK293T cell line exhibits low Wnt signaling activity, whereas HCT116 cells display moderate levels of Wnt activity due to a mutation in β-catenin. In contrast, SW620 cells have maximized Wnt signaling due to mutations in the *Adenomatous Polyposis Coltable 2* (APC) gene. A T-cell factor (TCF)/β-catenin-dependent reporter assay shows that *Pg*-CFS enhances Wnt signaling activity only in HCT116 CRC cells (Figures 1A; S1A); thus, we continued our study using this cell line. To test the specificity of the *Pg-*CFS effect, we used CFS derived from two control microbiota: *Escherichia coli (Ec),* which is a gram-negative bacterium that is part of the normal intestinal flora but can also cause intestinal and extraintestinal illnesses in humans, and *Clostridium butyricum* (Cb), a probiotic, an anaerobic bacterium that is a common commensal bacterium in the human and animal gut,^30^ shown to reduce cancer progression.^31^ As shown in Figure 1B, only the CFS of *P. gingivalis* enhanced the Wnt signal following 24 h of incubation. β-catenin, a key component of the canonical Wnt signaling pathway, is translocated to the nucleus upon activation, where it upregulates the expression of Wnt target genes. In addition, β-catenin promotes adherens junction formation by binding to E-cadherin and induces EMT when released from this complex.^32^ The *Pg*-CFS treatment led to a decrease in the interaction between E-cadherin and β-catenin (Figure 1C), which resulted in the accumulation and nuclear translocation of β-catenin (Figure 1D). Together, these findings suggest that secreted factors produced by *P. gingivalis* can enhance canonical Wnt signaling activity in CRC cells expressing moderate levels of Wnt signaling and induce the nuclear localization of β-catenin.

### The CFS of *P. gingivalis* affects cellular adhesion and induces the expression of EMT markers

**Figure 2.**
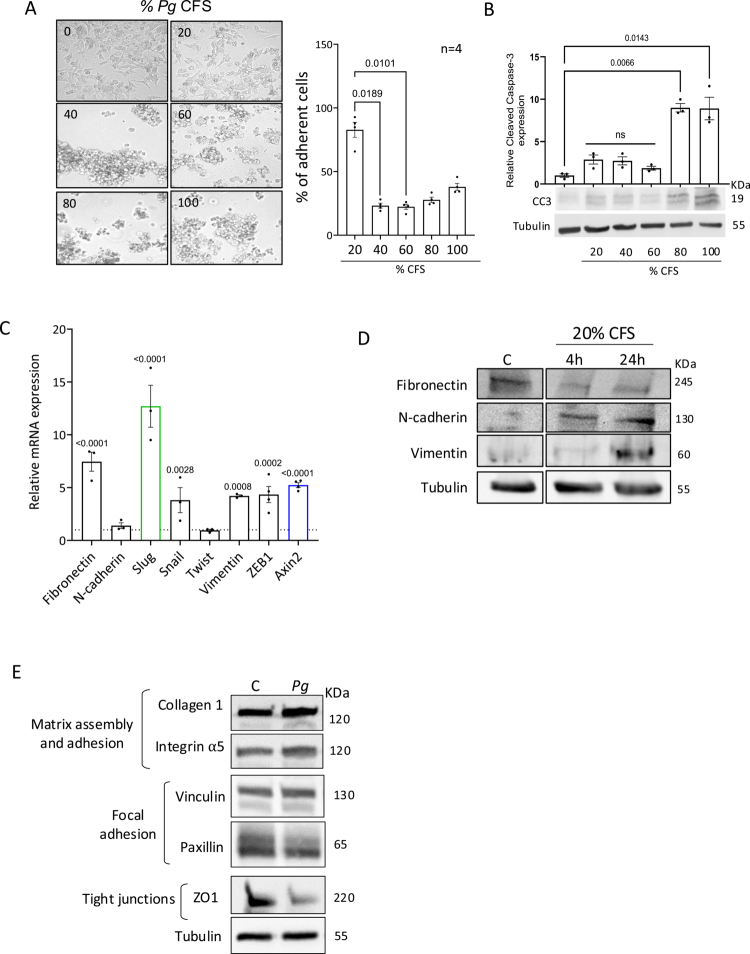
The CFS of *P. gingivalis* affects cellular adhesion and induces EMT markers. HCT116 cells were treated with increasing concentrations of *Pg*-CFS (0%–100%) for 24 h. (A) Cellular adherence was determined by Alamar blue analysis as described in the methods section. The results were normalized to the Wilkins broth control. Kruskal‒Wallis ANOVA was employed (*p*-value = 0.0031), and Dunn's multiple comparisons test was used. *p-*values are indicated in the graphs. (B) Cell lysates were extracted for cleaved caspase-3 WB analysis. The results were normalized to the Wilkins broth control. Kruskal‒Wallis ANOVA was employed (*p*-value = 0.0031), and Dunn's multiple comparisons test was used. *p*-values are indicated in the graphs. (C) HCT116 cells were treated with 20% *Pg*-CFS for 24 h. Next, the cells were subjected to quantitative PCR (qPCR) analysis for the indicated genes. Actin was used as a control. The results were normalized to the Wilkins broth control. A two-way ANOVA with Holm‒Šídák's multiple comparisons test was employed; *p*-values are indicated in the graphs. (D) HCT116 cells were treated with control and 20% *Pg*-CFS for 4 h or 24 h. The cells were then harvested and subjected to WB analysis using the indicated antibodies. (E) HCT116 cells were treated with control and 20% *Pg*-CFS for 24 h, and cell lysates were subjected to WB analysis using the indicated antibodies. Tubulin was used as a loading control.

To further decipher *P. gingivalis'*s mode of action, the cells were treated with different concentrations of the bacterial CFS. Following 24 h of incubation, the cells treated with at least 40% *Pg*-CFS displayed an aggregated, spherical morphology with reduced adherence and detached from the plate surface (Figure 2A). To determine cell viability following treatment, cleaved Caspase-3 analyses and Alamar blue staining were conducted (Figures 2B, S2), ruling out significant cell death at 20%–60% *Pg*-CFS concentrations (compared to the Wilkins broth control). Therefore, 20% and 40% *Pg*-CFS were used in the following experiments. Morphological changes and cellular detachment are coupled with EMT, and indeed, the expression levels of several EMT regulating-genes were upregulated following incubation with the *Pg*-CFS, most notably the expression of Slug (Figure 2C). Axin2, a Wnt target gene, is also implicated in EMT processes, and the correlation between Axin2 and Slug was previously shown both in lip development and ovarian cancer.^33^^,^^34^ The expression levels of various EMT proteins were also examined following treatment with the *Pg*-CFS (Figure 2D). The levels of both N-cadherin and vimentin were increased. In contrast, the expression levels of fibronectin were reduced, most likely as a result of protein degradation mediated by bacterial secreted proteases, which specifically degrade fibronectin to weaken epithelial adhesion and promote invasiveness.^35-38^*P. gingivalis* was shown to promote the invasive ability of oral cancer cells via the upregulation of matrix metalloproteinases (MMPs).^39^ However, no significant effects were detected in either Slug/Snail protein expression levels or Wnt activation in CRC cells when the MMP-specific inhibitor (GM6001) was used (Figure S3A and B). In addition, matrix assembly proteins such as collagen 1 and integrin α5, which are known to be degraded by MMPs, were not affected by the *Pg*-CFS (Figure 2E). Interestingly, the tight junction protein, ZO1, was significantly decreased following *Pg*-CFS treatment, which aligns with previous studies demonstrating that secreted *P. gingivalis* gingipains degrade ZO1.^40^

### The CFS of *P. gingivalis* increases the expression of Slug

**Figure 3.**
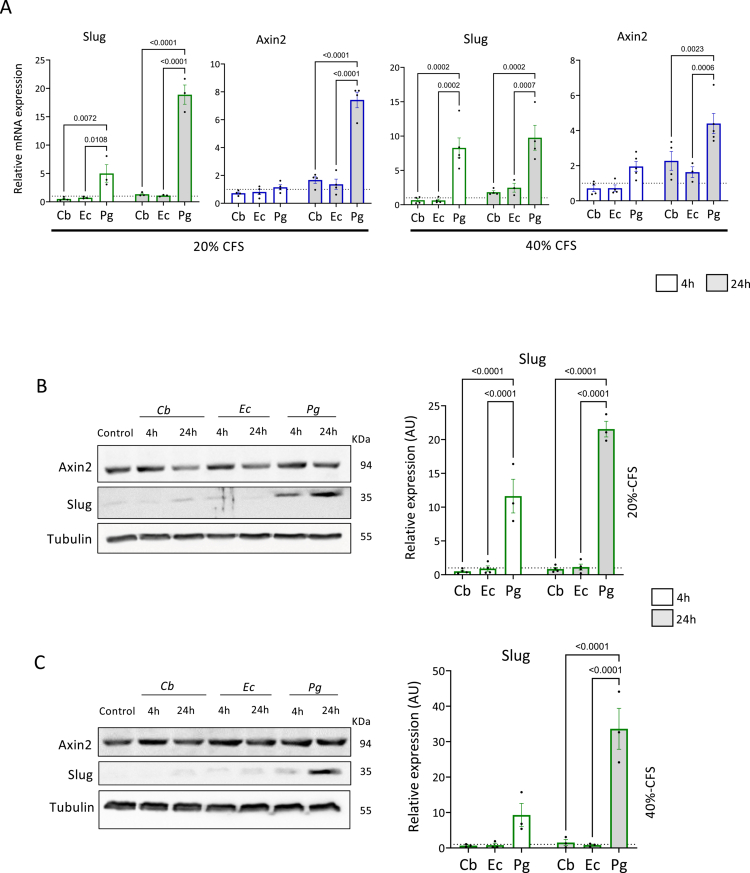
The CFS of *P. gingivalis* increases the expression of Slug. (A) HCT116 cells were treated with 20% (left) or 40% (right) CFS from *C. butyricum*, *E. coli,* and *P. gingivalis* bacteria for 4 h or 24 h and subjected to qPCR analysis for the indicated genes. Actin was used as a control. The results were normalized to the Wilkins broth control. A two-way ANOVA with Holm‒Šídák's multiple comparisons test was employed; *p-*values are indicated in the graphs. (B and C) HCT116 cells were treated with 20% (B) or 40% (C) CFS from *C. butyricum*, *E. coli,* and *P. gingivalis* bacteria for 4 h or 24 h. The cells were harvested and subjected to WB analysis using the indicated antibodies. Tubulin was used as a loading control. Band quantification was calculated and normalized to the Wilkins broth control using ImageJ software. The expression levels of Axin2 are shown in Figure S4. A two-way ANOVA with Holm‒Šídák's multiple comparisons test was employed; *p-*values are indicated in the graphs. Pg = *P. gingivalis*, Ec = *E. coli*, and Cb = *C. butyricum.*

To further elucidate the effects of *Pg*-CFS on the expression of Slug and Axin2, 20% and 40% CFS following short (4 h) and long (24 h) term incubation periods were used. The results show that, at the mRNA level, the *Pg*-CFS specifically increased the expression of both Axin2 and Slug compared to *C. butyricum* and *E. coli* controls (Figure 3A). Interestingly, the effect on Slug was detected in both CFS concentrations as early as 4 h after treatment, whereas Axin2 upregulation was observed only later (Figure 3A). Next, the effect of the bacterial CFSs on Slug and Axin2 protein levels was examined. Although the *Pg*-CFS strongly induced the expression of the Slug protein (in HCT116 cells, Figure 3A, but not in HEK293T cells, Figure S1B and C), Axin2 protein levels were unchanged regardless of the CFS concentration or incubation time (Figures 3B,C; S4). Previous findings also showed that Wnt-3A stimulation may induce Axin2 mRNA transcription without elevating protein levels, suggesting that Axin2 participates in a negative feedback loop that could limit the duration or intensity of Wnt signaling.^41^ Additionally, partial redundancy with Axin1, or the possibility that Axin2, similarly to Axin1, is directly degraded by bacterial gingipains that may be internalized by host cells, may explain this finding^40^^,^^42^. Although the effect of the *Pg*-CFS on Snail mRNA, another established EMT marker, was relatively moderate (Figures 2C, S5A), the increase in Snail’s protein expression correlates with that of Slug in both *Pg*-CFS concentrations (Figure S5B and C). Interestingly, on the mRNA levels, the induced expression of Snail at 20% *Pg*-CFS was similar to that of Axin2 (after 24 h of treatment), while 40% *Pg*-CFS led to early upregulation of Snail, much like the effect on Slug (Figures 2, S5A). Together, these results suggest that secreted products from *P. gingivalis* influence EMT markers, including Slug, Snail, and Axin2, although the underlying mechanism may differ for each gene.

**Figure 4.**
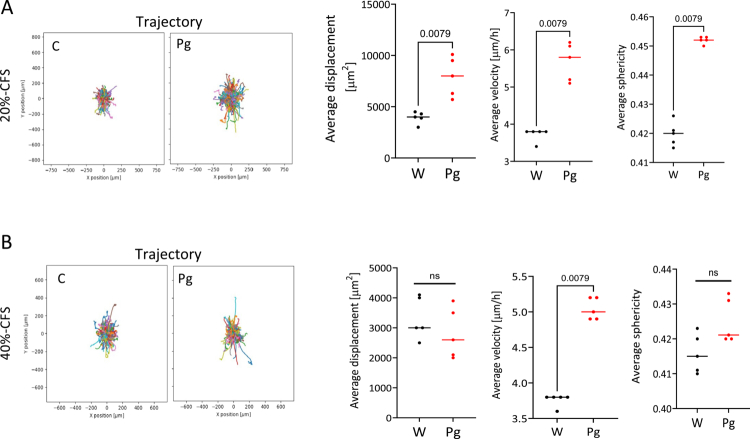
The CFS of *P. gingivalis* affects the cells' spatial properties (A and B). HCT116 cells were confluently seeded in 96-well plate. A wound healing scratch was applied, followed by incubation with 20% (A) or 40% (B) *Pg*-CFS or the control for 48 h with Incucyte analysis. Morphology and motility parameters, such as the cellular trajectory (left) and displacement (right), of the cells in the scratch area were analyzed using IMARIS with the Imaris surface mode.^44^ A non-parametric Mann‒Whitney test was employed; the *p*-value is indicated on the graph.

One major parameter of the EMT process is cell motility. Thus, scatter and scratch assays were used to examine the effect of the *Pg*-CFS on cellular movement. The cells were treated for 48 h with *Pg*-CFS or the control and monitored using Incucyte time-lapse imaging analysis to analyze multiple cellular morphology and motility parameters simultaneously. The results show that cells' trajectory, displacement, velocity, and sphericity properties were increased following 20% *Pg*-CFS treatment (Figure 4A,B). Interestingly, augmented sphericity corresponds with amoeboid behavior, a stage in the EMT process that characterizes more aggressive and rapidly moving cells with reduced interaction with the extracellular matrix (ECM).^43^ Anchored cells exposed to 40% *Pg* CFS showed a reduced response, indicating that these remaining adherent cells were less affected by the bacterial secreted factors. Morphokinetic analysis (using the results from this study) is shown in Supplementary Table 1, which places the cells on a motility continuum from epithelial to amoeboid motility, with specific references to the experimental results.

### *P. gingivalis* CFS affects the Hippo signaling pathway

**Figure 5.**
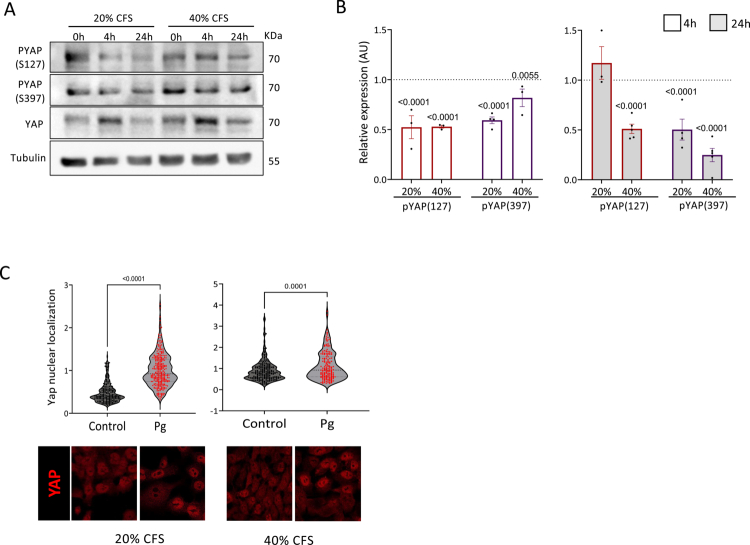
The CFS of *P. gingivalis* affects the Hippo signaling pathway. (A and B) HCT116 cells were incubated with 20% or 40% *Pg*-CFS for 4 h or 24 h. Next, the cells were subjected to WB analysis using the indicated antibodies. Tubulin was used as a loading control (A). Band quantification was measured by ImageJ software (B). The results were normalized to the Wilkins broth control. A two-way ANOVA with Holm‒Šídák's multiple comparisons test was employed; *p-*values are indicated in the graphs. (C) HCT116 cells were incubated with 20% or 40% *Pg*-CFS for 4 h or 24 h, then stained with anti-Yap antibody and visualized using confocal microscopy. The nuclear localization of Yap was analyzed from 7 independent fields of each sample using QuPath software. An unpaired *t*-test with Welch's correction was employed; the *p*-value is indicated in the graph.

The *Pg*-CFS upregulation of Slug preceded that of Axin2, suggesting that the increase in the expression of Slug may not result from Wnt signaling activation. Thus, the Hippo–Yap/Taz signaling pathway was examined, as both Slug and Axin2 are targets of this pathway.^45^^,^^46^ Activated Hippo signaling leads to the phosphorylation of Yap on S127 or S397 residues, resulting in its cytoplasmic retention or proteasomal degradation, respectively.^47^ The effects of the *Pg*-CFS on Yap expression, phosphorylation, and cellular localization were examined. The results indicate that following 4 h of incubation, the expression levels of Yap were increased, whereas the phosphorylation of both S127- and S397-pYAP was decreased (Figure 5A,B, left panel). These findings suggest that the Yap protein may be sequestered from the cytoplasm to the nucleus, where it can upregulate the expression of Slug. Immunofluorescent staining demonstrated that, indeed, the YAP protein accumulated in the cells' nuclei (Figure 5C). In these experiments, the effect of 20% *Pg*-CFS was more prominent compared to the 40% CFS, which correlates with the Slug and Axin2 enhanced expression levels (Figure 3). Interestingly, following 24 h of incubation with the *Pg*-CFS, the increase in the phosphorylated S397-pYAP was accompanied by decreased total Yap levels, indicating protein degradation (Figure 5A and B-right panel). These results suggest that the initial upregulation of Slug may be associated with the activation of the Hippo pathway, and later maintained by Wnt signaling activity.

### Gingipain proteases secreted by *P*. gingivalis affect cellular adhesion

**Figure 6.**
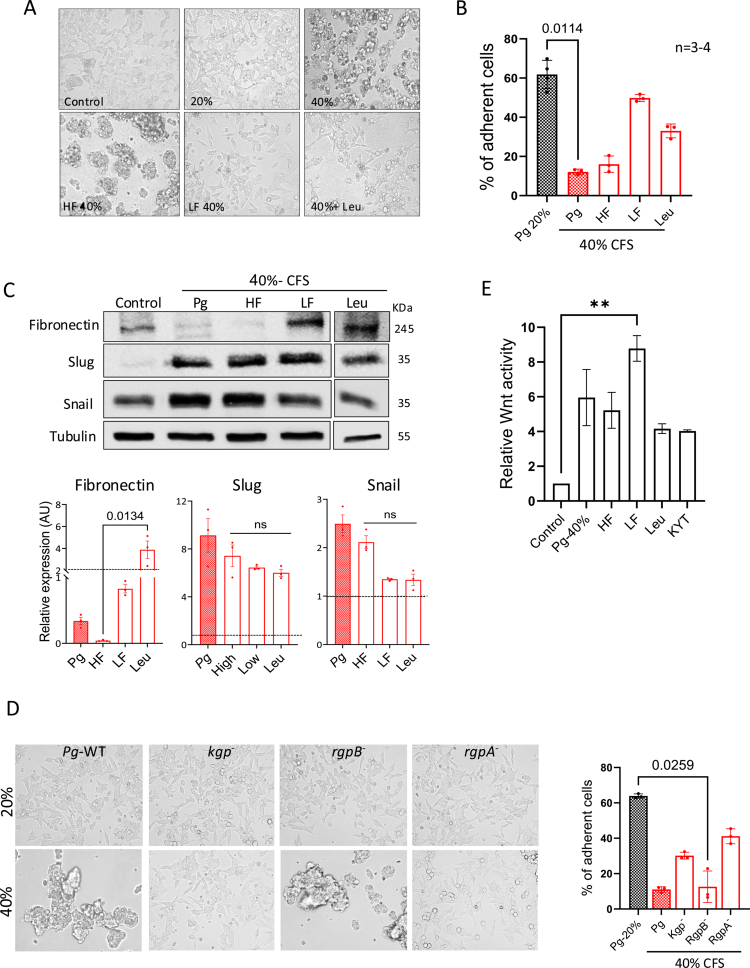
Gingipain proteases secreted by *P. gingivalis* affect cellular adhesion. (A and B) HCT116 cells were treated with *Pg*-CFS, high (HF) or low (LF) molecular weight fractions of *Pg*-CFS (above or below 10 kDa, respectively), the protease inhibitor Leupeptin (1 mM; Leu), or a control for 24 h (A). Cellular adherence was determined by Alamar Blue analysis (B). The results were normalized to the Wilkins control. Kruskal‒Wallis ANOVA with Dunn's multiple comparisons tests were employed separately for the *Pg* treatments at 20% and 40% (*P*-value < 0.0001) and for the *Pg* 40% treatment (*P*-value = 0.0002). *P*-value is indicated in the graph. (C) HCT116 cells were treated with the specified CFSs for 24 h and subjected to WB analysis using the indicated antibodies. Tubulin was used as a loading control. Band quantification was calculated for the indicated proteins (Fibronectin, Slug, and Snail) using ImageJ software. The dashed line depicts the control values (set as 1). Kruskal‒Wallis ANOVA with Dunn's multiple comparisons test was employed. *p*-values are indicated on the graphs. (D) HCT116 cells were treated with CFS obtained from *P*. *gingivalis* wild-type, *kgp^−^, rgpB^−^*, and *rgpA*^−^ mutant strains. Cellular adherence was visualized using light microscopy and quantified by Alamar Blue analysis. Kruskal‒Wallis ANOVA with Dunn's multiple comparisons test was employed. *p*-value is indicated in the graph. (E) HCT116 cells were transfected with the TOPFLASH/FOPFLASH reporter vectors following 24 h incubation with the indicated CFS (high (HF) or low (LF) molecular weight fractions of Pg-CFS, 1 mM Leupeptin, 0.5 mM KYT-41). The cells were harvested and subjected to a Luciferase assay. The results were normalized to the Wilkins broth control. Kruskal‒Wallis ANOVA with Dunn's multiple comparisons test was employed. *p*-value is indicated in the graph.

To identify the bacterial factors that may contribute to our findings, the *Pg*-CFS was examined by mass spectrometry. The three proteins with the highest frequency detected were the gingipain proteases *Rgp*A, *RgpB*, and *Kgp* (Supplementary Table 2). These cysteine proteases have been previously shown to disrupt cellular adhesion by degrading adhesion proteins, thereby weakening tissue integrity and facilitating bacterial invasion. To define the effects of the secreted proteases, the CFS was divided into a low (<10 kDa) molecular weight fraction (LF) and a high (>10 kDa) molecular weight fraction (HF), which contains the gingipain proteases. As shown above (Figure 2A), augmented cell detachment was clearly observed following incubation with 40%-CFS (Figure 6A,B). Similarly to the 20% CFS, the low-molecular-weight fraction (LF) did not affect cell adherence. In contrast, the high molecular weight fraction (HF) promoted cell detachment, suggesting that the gingipain protease found in this fraction mediates this effect. The addition of Leupeptin, which inhibits cysteine proteases, partially reversed the detachment effect, further supporting the hypothesis that the *Pg*-secreted gingipains affect CRC cell adhesion. Both the *Pg-*CFS high and low fractions had a similar impact on Slug as the complete *Pg*-CFS, which Leupeptin only partly inhibited (Figure 6C). Additional concentrations of Leupeptin demonstrated a small effect on *Pg*-mediated Slug upregulation (Figure S6). Interestingly, the inhibitory effect of Leupeptin led to a significant elevation in the expression levels of the Fibronectin protein, which is in agreement with previous reports demonstrating that gingipains or other bacterial proteases promote its proteolysis.^35-37^ To determine the involvement of specific gingipains, we used *P. gingivalis* mutant strains defective for each protease (*kgp*^−^, *rgpB^−^,* and *rgpA*^−^). CFS derived from each mutant and the wild-type control were used to treat the cells at concentrations of 20% and 40%, respectively. Our results suggest that both *Kgp* and *RgpA* may be involved in mediating the detachment effect driven by the *Pg*-CFS (Figure 6D). We next tested the effects on Wnt activity. Neither Leupeptin nor blocking the bacterial gingipains using KYT-41, a potent inhibitor of Arg- and Lys-gingipain,^48^ reduces the bacterial-induced Wnt signal (Figure 6E), suggesting that the gingipains are not responsible for the *Pg*-CFS-induced activation of the Wnt signal. A slight yet significant increase in the Wnt signal was detected when the cells were treated with the low-molecular-weight fraction, implying that other secreted metabolites may prompt the *P. gingivalis* effect on Wnt activity.

### The effects of the *P. gingivalis*-CFS are mediated via H_2_S

**Figure 7.**
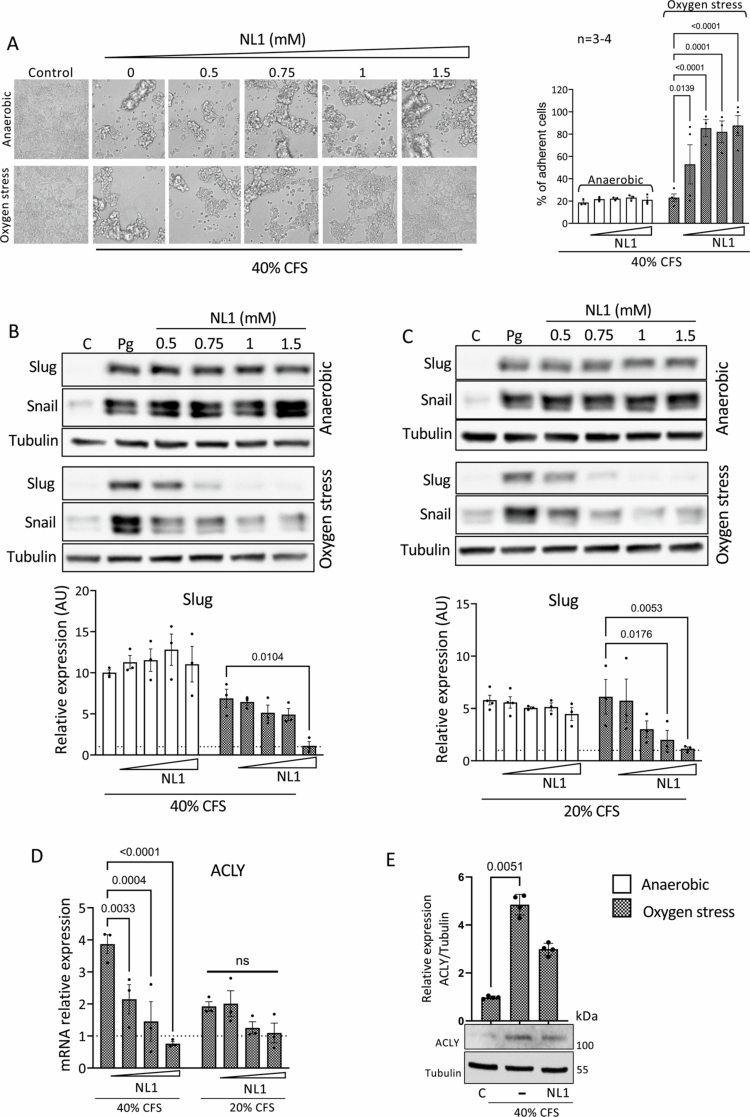
The effects of the *P. gingivalis*-CFS are mediated via H_2_S. *P. gingivalis* bacteria were cultured anaerobically or under oxygen stress for 72 h in the absence or with increasing concentrations of the NL1 inhibitor (0.5–1.5 mM). The bacterial CFSs were then used to treat HCT116 cells for 24 h, and cellular adherence was determined by Alamar Blue analysis (A). The results were normalized to the Wilkins control. A two-way ANOVA with Holm‒Šídák's multiple comparisons test was employed; *p*-values are indicated in the graphs. (B and C) HCT116 cells were treated with 20% (C) or 40% (B) *Pg*-CFS (cultured anaerobically or with oxygen stress) with the indicated NL1 concentration for 24 h, then subjected to WB analysis using Slug and Snail antibodies. Tubulin was used as a loading control (upper panels). Band quantification was measured using ImageJ software (lower panels). A two-way ANOVA with Holm‒Šídák's multiple comparisons test was employed; *p*-values are indicated in the graphs. (D) *P. gingivalis* cultured under oxygen stress for 72 h were treated with increasing concentrations of the NL1 inhibitor (0, 0.5, 1, or 1.5 mM). The bacterial CFSs were used to treat HCT116 cells for 24 h. ACLY gene expression was detected by qPCR analysis, using actin as a control. The results were normalized to the Wilkins broth control. A two-way ANOVA with Holm‒Šídák's multiple comparisons test was employed; *p*-values are indicated in the graphs. (E) CFS from *P. gingivalis* cultured under oxygen stress for 72 h and treated with 1 mM NL1 was used to treat HCT116 cells for 24 h. ACLY protein expression was detected by WB analysis. Tubulin was used as a loading control. Band quantification was established using ImageJ software. Kruskal‒Wallis ANOVA with Dunn's multiple comparisons test was employed. *p*-value is indicated in the graph.

Another secreted *P. gingivalis* virulent factor is H_2_S. To assess whether the effects of the bacterial CFS can be attributed to H_2_S, we used the CSE-specific inhibitor NL1, which has been shown to eliminate most H_2_S production in *S. aureus* and *P. aeruginosa* bacteria.^49^ As expected, NL1 also eliminated the production of H_2_S by *P. gingivalis* (Figure S7A). *P. gingivalis* was cultured under anaerobic (optimal growth) or oxygen stress conditions in the absence or in the presence of increasing concentrations of the NL1 inhibitor (0.5–1.5 mM). NL1 added to bacteria grown under anaerobic conditions did not alter the CFS effect on cell adherence (Figure 7A; upper panels), except at very high concentrations (not shown). However, CFS obtained from bacteria exposed to oxygen stress and treated with NL1 (0.5–1.5 mM) significantly restored cellular adhesion (Figure 7A; lower panels). A parallel experiment conducted with 20% *P. gingivalis* (+/− oxygen stress; NL1 treatment) did not affect cell detachment, as predicted (Figure S7B). Additionally, we examined the effects of NL1 inhibition on cellular adhesion using CFS from wild-type *P. gingivalis* and the *kgp*^−^, *rgpB*^−^, and *rgpA*^−^ mutants cultured under oxygen stress (Figure S8). These results indicate that the two bacterial virulence factors do not synergize. We then examined the effect of the NL1 treatment on *P. gingivalis–*mediated upregulation of Slug and Snail. The increase in the expression of both proteins following *Pg*-CFS treatment was blocked by the NL1 inhibitor, regardless of the CFS concentration, but only when the bacteria were exposed to oxygen (Figure 7B,C). These results suggest that H_2_S produced by stressed bacteria contributes to cell detachment and increased EMT. Interestingly, endogenously produced H_2_S in cancer cells promotes EMT, involving, at least in part, the upregulation of ATP citrate lyase (ACLY) protein expression.^50^ Indeed, ACLY was demonstrated to stimulate colon cancer metastasis *in vitro* and *in vivo*. The mechanism of action involves the stabilization of β-catenin and its translocation to the nucleus, which subsequently promotes its transcriptional activity and the cancer cells' enhanced migration and invasion abilities.^51^ However, Wnt activity was unchanged following NL1 treatment (Figure S9), suggesting that additional bacterial factors may contribute to the upregulation of Wnt signaling. Treating the cells with 40% *Pg*-CFS led to increased expression of ACLY (Figure 7D,E), which may account for the increase in EMT markers. The addition of NL1 blocked the upregulation of ACLY in a dose-dependent manner, reinforcing the notion that bacteria-produced H_2_S affects the cell’s EMT expression pattern by mediating the ACLY protein.

## Discussion

Emerging data highlight the effects of the gut microbiome on colon cancer progression, specifically the imbalance of the bacterial microbiota, which is strongly associated with CRC. However, the various mechanisms by which gut microbiota dysbiosis contributes to CRC development are still being discovered. *P. gingivalis*, an oral pathogen, is enriched in CRC and enhances cancer tumorigenesis via different mechanisms.^22^^,^^25-29^

In the present study, our goal was to mechanistically dissect the biochemical and signaling activities of *P. gingivalis* secretome. The usage of bacterial CFS, rather than the bacteria themselves, enabled us to determine the specific effects of distinct secreted factors by utilizing CFS fractionation, protease or enzyme inhibitors, and deficient mutant strains. Our results establish a set of secreted factors that can now be systematically evaluated in future studies using *P. gingivalis* bacterium.

We demonstrate that the *Pg-*CFS induces Wnt/β-catenin signaling, a major driving force behind the development of CRC,^52^ in CRC cells harboring moderate levels of Wnt signaling. In addition to its function as a core component of the canonical Wnt pathway, β-catenin is part of the E-cadherin/β-catenin complex that maintains the integrity of epithelial cell‒cell contact. Disruption of these cell‒cell complexes releases β-catenin, which can then enter the nucleus and transduce the Wnt signal.^53^ Indeed, incubation of the CRC cells with the *P. gingivalis-*CFS reduces the E-cadherin/β-catenin interaction and increases β-catenin nuclear localization. This is in line with recent findings showing that infecting oral squamous cell carcinoma cells with *P. gingivalis* increased the expression of nuclear *β*-catenin.^54^ *P. gingivalis* has been previously shown to induce EMT in various cell types.^55-57^ Our results show that incubating CRC cells with 40%–60% *Pg-*CFS led to reduced cellular adherence coupled with a more spherical morphology without altering cell viability. In addition, the treated cells demonstrated increased expression of EMT markers, most notably Slug,^58^ which is also a Wnt target gene.^59^ However, Slug upregulation was also observed when the cells were treated with 20% CFS, a concentration that did not induce cell detachment. The levels of Axin2, another Wnt target gene shown to promote the Wnt/β-catenin initiated EMT program in CRCs, ^60^ were increased only at the mRNA level, further demonstrating the intricate relationship between the two pathways. The increased expression of various EMT genes following *Pg*-CFS treatment was accompanied by advanced cell displacement, increased motility, decreased sphericity, and enhanced cellular suspension. Cells that transition from adherent to suspension exhibit cancer features, such as EMT, that are related to metastasis.^61^ EMT proceeds through a spectrum of intermediate states and can be classified into four main types: (1) partial-EMT (pEMT), which involves a mixture of epithelial and mesenchymal markers and has a greater migratory ability, allowing it to detach from its original tissue found in invasion regions, and has high metastatic abilities. In this stage, Slug plays a dominant role^62^^,^^63^ (2 and 3) intermediate to extreme EMT, leading to the expression of mesenchymal markers. (4) The mesenchymal stage or an ameboid EMT stage is characterized by high mechanoplasticity, migration, and metastasis capabilities.^64^ Amoeboid migration is a specialized mode of movement employed by specific cancer cells, allowing them to move faster through the ECM with great flexibility.^65^ Cells that adopt amoeboid migration usually present a combination of high displacement and sphericity that can be explained by their weak connections to the ECM and their ability to transfer between tissues.^43^^,^^65^ The data suggest that *Pg*-CFS drives a pEMT in HCT116 cells, biasing them toward an invasive, quasi-amoeboid motility phenotype. *Pg*-CFS induces a hybrid EMT/amoeboid phenotype characterized by an increase in Slug/Snail expression, YAP activation, weakened cell adhesion, and enhanced motility, thereby promoting an aggressive migratory behavior. *P. gingivalis* has been shown to interfere with epithelial cell proliferation and migration through mechanisms involving bacterial tyrosine phosphatases, such as Ltp1.^66^ Furthermore, prolonged exposure to *P. gingivalis* has been shown to promote EMT-like, in oral keratinocytes and oral squamous cell carcinoma cells.^57^^,^^67^^,^^68^ These findings suggest that *P. gingivalis*, through its secreted factors, can significantly impact the motility and EMT of epithelial cells, potentially contributing to cancer progression and metastasis.

*P. gingivalis* secretes several virulence factors, including lipopolysaccharides (LPS), gingipains, tetratricopeptide repeat sequence protein, extracellular polysaccharides, the hemoglobin uptake system,^69^ as well as short-chain fatty acids such as butyrate.^29^ Mass spectrometry analysis revealed that the most abundant proteins in the *P. gingivalis*-CFS were the three gingipain proteases (consisting of two types of arginine-specific proteases- *RgpA* and *RgpB*, and a lysine-specific protease – *Kgp*), that play a significant role in periodontal disease by disrupting cellular adhesion. This finding is in line with studies that have shown that gingipains can be secreted and released into the extracellular environment, presented on the bacterial surface, found in culture supernatants and internalized into host cells, where they play important roles, such as in degrading host proteins and inducing cell migration, immune system evasion, bacterial adhesion, and biofilm formation.^40^^,^^70-75^ Gingipain proteases impair the integrity of the tissue barrier by breaking down key adhesion molecules, thus facilitating bacterial invasion and inflammation.^76^ The protease's effect on CRC cells was examined using CFS fractionation and gingipain-deficient mutant strains. The results show that the CFS's high molecular weight fraction (HF), which contains secreted proteases, is primarily responsible for the detachment effect. The addition of Leupeptin, which inhibits cysteine proteases,^77^ essentially reversed the effect of 40% *Pg*-CFS and restored cellular adherence. This observation correlates with previous findings showing that *P. gingivalis* outer cellular vesicles induce epithelial cell detachment in a gingipain-dependent manner.^78^ Extracellular Arg-x- (*Rgp*) and Lys-x (*Kgp*) specific cysteine proteinases are considered important virulence factors and pathogenic markers for *P. gingivalis* ^79^, and mutant strains lacking *Kgp*, *RgpA*, or *RgpB* are crucial tools for studying bacterial effects. We used three mutant strains (*kgp*^−^, *rgpA*^−^*,* and *rgpB^−^*) to verify the effects of secreted gingipains on cell adherence. Our results indicate that *Kgp* and *RgpA* are most likely involved in mediating the cellular detachment induced by *Pg*-CFS treatment. *P. gingivalis* was shown to produce extracellular complexes of proteinases and adhesins, designated the *RgpA-Kgp* complexes (or high-molecular-weight gingipains), and a prolonged interaction of the host immune system with these complexes is believed to be a significant factor in tissue destruction in chronic periodontitis.^80-82^ It was also shown that the *RgpA-Kgp* complex efficiently cleaves pro-uPA, and plasminogen, yielding active uPA and plasmin, which in turn may promote cell migration.^83^^,^^84^ *Kgp* and *RgpA* were also found to mediate the degradation of Plasminogen Activator Inhibitor-1.^85^ Taken together, it is plausible that *Kgp* and *RgpA* proteases work synergistically to impair cellular adherence, leading to increased cell detachment as seen in the high molecular fraction treatment. As expected, only the *Pg*-CFSs HF led to the degradation of Fibronectin, which was abolished when Leupeptin was added. However, both LF and HF induced the expression of the EMT genes Snail and Slug, and Leupeptin only partly reversed this phenotype. Moreover, the low-molecular-weight fraction (LF) enhanced Wnt activity beyond the effect of *Pg*-CFS. Together, these results suggest that secreted gingipain proteases are partially involved in cell adhesion and EMT properties. Nevertheless, other bacterial factors, most likely small molecules or metabolites, can mediate gene expression and Wnt signaling activity.

A vital defense system and a virulence factor are shared by several bacterial species and utilized by *P. gingivalis* is H_2_S,^86^^,^^87^ which protects bacteria from oxidative stress.^24^ H_2_S is a key signaling molecule in cancer biology due to its unique chemical properties and ability to alter proteins.^88^ Colonic bacteria are responsible for generating vast quantities of H_2_S, which exceed the normal range.^89^ Indeed, several studies have found that H_2_S plays an important role in CRC^90-95^ and can promote cancer cell growth, stimulate cellular bioenergetics, enhance angiogenesis, induce dedifferentiation, invasion, and metastasis, and confer chemotherapeutic resistance.^96^ Additionally, H_2_S is a crucial gasotransmitter involved in various vital biological functions, including inflammation, gut motility, oxidative stress, ulcer healing, and vascular tone.^89^ During migration through the oral cavity or other organs, *P. gingivalis* encounters oxidative stress^97^ and produces large amounts of H_2_S as a response.^98^ To test the involvement of H_2_S in our findings, we examined the effects of inhibiting H_2_S production, using NL1, a potent H_2_S biosynthesis inhibitor.^49^ The results demonstrate that impeding H_2_S production under oxidative stress conditions completely blocked the inducing effect of both 20% and 40% *P. gingivalis*-CFS on Slug and Snail expression, consistent with findings showing that H_2_S biosynthesis inhibitors reduce CRC EMT and migration properties.^50^ Inhibiting H_2_S biosynthesis did not reverse the bacteria's effects under anaerobic conditions. Despite the colonic environment being predominantly anaerobic, approximately 5% oxygen can be found in areas of the epithelial lining,^99^ due to tissue oxygen diffusion and metabolism by the epithelium. While *P. gingivalis* is classically defined as an obligate anaerobe, and several studies have shown that it can also survive and grow under low-oxygen (6%–10%) conditions.^100-102^ Although hypoxia is common within the CRC microenvironment, both tumor cells and the microenvironment exhibit steep oxygen gradients, including perivascular niches that are relatively better oxygenated^103^ and can support bacterial growth. Thus, in colorectal inflammation and cancer, *P. gingivalis* may be exposed to oxygen, particularly at invasive fronts or adjacent to blood vessels, and possesses mechanisms that enable its survival in such niches. The ATP citrate lyase (ACLY), a pivotal enzyme in lipid metabolism, was shown to be upregulated by H_2_S and to promote EMT in CRC cells through interaction with β-catenin, thus favoring its nuclear translocation and the expression of Wnt downstream target genes.^51^^,^^104^ Our results show that in addition to inducing the EMT markers, H_2_S at 40% CFS leads to a significant increase in ACLY expression, which was blocked by the NL1 inhibitor. Several studies have highlighted the involvement of *P. gingivalis* in CRC, demonstrating that various cellular and secreted bacterial elements function through distinct mechanisms to affect different stages of CRC. For example, *P. gingivalis* accelerates CRC development by enhancing the adhesion of bacteria to the colonic epithelium.^22^^,^^26-28^ The gingipains can also enable bacterial evasion from the host immune response by cleaving surface receptors and inducing cytokine degradation.^80^^,^^105^ Other pathogenic mechanisms involve induced cellular senescence through the secretion of the short-chain fatty acid butyrate.^29^ In these cases, similar to the current study's findings, the bacteria accelerate and enhance the tumorigenic process. Numerous publications that discuss the distinct functions of H_2_S and gingipains report that both can affect cell mobility, motility, and adherence.^106-112^ Our results indicate that there is no synergistic effect between the functions of gingipain proteases and the H_2_S defense system. The *rgpB*^−^ mutant showed a similar phenotype to the wild-type *P. gingivalis,* and the *kgp*^−^ *and rgpB*^−^ mutants reversed the phenotype with and without NL1 treatment. Supporting this finding is the observation that H_2_S is produced by both wild-type and gingipain-deficient *P. gingivalis* mutants leads to the upregulation of the proinflammatory cytokine IL-8.^113^

Another small molecule, the short-chain fatty acid (SCFA) butyrate, is secreted by *P. gingivalis*, has been shown to promote CRC development by inducing cellular senescence and pro-inflammatory gene expression.^29^ While SCFAs are generally regarded as colonic anti-inflammatory metabolites, butyrate appears to exert context-dependent effects. In the setting of dysbiosis and tumorigenesis, butyrate may induce cytokines typically associated with inflammation, including IL-1-related pathways, and promote senescence-associated secretory phenotypes that reshape the tumor microenvironment. Thus, in contrast to the canonical anti-inflammatory role attributed to SCFAs in healthy tissue, *Pg*-secreted butyrate may contribute to a pro-tumorigenic inflammatory environment. Whereas butyrate reshapes the tumor microenvironment primarily through senescence and inflammatory modulation, H₂S enhances invasive behavior by weakening epithelial adhesion and promoting EMT and amoeboid motility. Together, these findings support a model in which *Pg*-derived metabolites exert distinct yet complementary tumor-promoting effects. Nevertheless, as the present study is limited to the effects of *Pg-*CFS on CRC cells, *in vivo* validation in animal models, as well as examination of clinical samples, is required to confirm the physiological relevance of our findings.

Our data imply that secreted *P. gingivalis* factors stimulate Wnt signaling through an alternative mechanism involving gingipains, H_2_S, and most likely additional components. One possibility is that *P. gingivalis* can modify E-cadherin expression levels and localization, followed by the sequestering of β-catenin from the cell membrane to the nucleus,^55^ which is consistent with the induction of an EMT phenotype. Another possibility is that the *Pg*-lipopolysaccharides stimulate the Wnt/β-catenin pathway by inducing LRP6 phosphorylation^114^ and triggering p38 MAPK activation.^115^ Importantly, short-chain fatty acids, especially butyrate, can activate Wnt/β-catenin signaling by inhibiting histone deacetylases, thereby enhancing β-catenin stability and nuclear retention.^116^ Our finding that factors in the low-molecular-weight secretome fraction enhance Wnt signaling may support this notion.

In conclusion, we demonstrate that secreted factors of the *P. gingivalis* bacterium alter major cell characteristics in a time- and concentration-dependent manner. These changes affect the morphology, adherence, and motility of cancer cells, thereby contributing to potential tumorigenic traits. Our findings also illuminate the role of the H_2_S-mediated bacterial defense system in the host cell response. Both endogenous H_2_S in colonic epithelial cells and exogenous H_2_S in the intestinal lumen contribute to the onset and progression of CRC. suggesting novel avenues for anti-cancer therapy development that may be mediated through the ACLY protein. In addition, a recent paper has demonstrated that PKM2 sulfhydration by H_2_S inactivates PKM2 activity to promote tumorigenesis and that inhibiting this process could be a potential therapeutic approach for targeting cancer metabolism.^117^ Future work should also investigate the interplay between *P. gingivalis* secreted metabolites, bacterial vesicles, and the host immune microenvironment to better understand how oral–gut microbial crosstalk promotes CRC development and progression.

## Materials & methods

### Cell culture and bacteria

The human embryonic kidney cell line HEK293STF, the human CRC cells HCT116, SW620 (which originate from the same individual as SW480 cells) and the mouse fibroblast cell line L-WRN (medium containing Wnt3a, R-spondin 3, and Noggin that activates the Wnt pathway) were cultured in Dulbecco's modified Eagle's medium (Biological Industries) supplemented with 10% fetal bovine serum (Sigma) and 100 units/ml penicillin/streptomycin (Biological Industries, Kibbutz Beit HaEmek, Israel). All the cells were maintained at 37 °C in a humidified atmosphere of 5% CO_2_. The bacteria *C. butyricum* and *P. gingivalis* ATCC 33277 were cultured in a reduced Wilkins–Chalgren broth (Thermo Fisher, Oxoid) at 37 °C in an anaerobic jar using gas packs (Thermo Fisher, Oxoid). The bacterium *E. coli* DH5α was cultured aerobically in reduced Luria-Bertani broth at 37 °C. The *P. gingivalis* mutant strains *kgp*^−^, *rgpA*^−^, and *rgPB*^−^ were grown in Wilkins Broth for 96 h under anaerobic conditions, and the CFS was collected similarly to the wild-type bacteria.

### Bacterial cell-free supernatant (CFS) preparation

*C. butyricum* and *P. gingivalis* were thawed into reduced Wilkins–Chalgren broth and cultured anaerobically overnight. Next, bacteria were transferred into a fresh Wilkins–Chalgren broth and incubated overnight. This step was repeated twice. Finally, at OD_600_ 1.8 (96 h for *P. gingivalis* and 72 h for *C. butyricum*), the bacteria were centrifuged for 1 h at 4500 × *g* at 4 °C, and the supernatants were filtered twice using a 0.22 µm pore size filter to generate the bacterial CFS. The bacterial CFS were aliquoted and stored at −20 °C. *E. coli* bacteria were thawed into reduced Wilkins–Chalgren broth and cultured aerobically overnight. Next, the bacteria were transferred into fresh Wilkins–Chalgren media and cultured at 37 °C until an optical density (OD_600_) of 1.8 was reached. Then, the bacterial CFS was collected, as mentioned before. Bacteria viability at OD_600_ 1.8 was confirmed by Propidium Iodide staining (PI) as shown in Figure S10.

### L-WRN conditioned media preparation

The conditioned media (CM) containing Wnt-3a, R-spondin, and Noggin were prepared according to the manufacturer's instructions, with some volume adjustments and minor modifications. Shortly thereafter, the cells were grown for 3 d until they reached full confluency, after which they were incubated for 24 h in fresh growth medium. Twenty-four hours later, the media was collected and centrifuged, and the supernatant was stored at 4 °C. Four days later, at the end of the 4th batch collection, the total CM volume was filtered through a 45 µm pore size filter, aliquoted, and stored at −20 °C.

### Plasmids and materials

The pEGFPC1 (Clontech) plasmid was used for the expression of GFP. The Wnt-responsive TCF-dependent luciferase plasmids pTOPFLASH (which includes multiple copies of wild-type TCF-binding sites linked upstream of a luciferase reporter gene) and pFOPFLASH (which contains mutated versions of the formerly described TCF-binding sites and thus serves as a negative control to monitor Wnt signal specificity) were kindly provided by H. Clevers and were described previously.^118^ pSV40-Renilla (SV40-Rnl), which was used as an internal normalization control for transfection efficiency, was purchased from Promega (Madison, WI, USA). GM6001, CC1010-K; Millipore. KYT-41, 4523-V; Pepta Nova.

To inhibit the gingipain proteases, 1 mM leupeptin (ENCO) was incubated with *P. gingivalis*-CFS at room temperature for 20 min before being applied to the cells for 24 h, followed by western blot analysis.

To inhibit the bacterial CSE enzyme, *P. gingivalis* bacteria were cultured with the indicated concentrations of the NL1 inhibitor (generously provided by Evgeny Nudler's laboratory) for 72 h before centrifugation.

### CFS fractionation

Bacterial CFS was fractionated using 10 kDa MWCO filter columns (Cytiva). The columns were washed twice with a column volume of sterile PBS. Then CFS was added to the column and centrifuged twice at maximum speed for 6 min at 20 °C. Next, the CFS from the higher fraction was diluted with Wilkins broth to reach the required volume (20% or 40%, respectively). Finally, CFS from the low fraction, as well as the diluted high fraction, were incubated with CRC cells for 24 h before being subjected to the indicated analysis.

### H_2_S indication

The gaseous fraction of H_2_S was measured by Lead Acetate Whatman indicator papers (Cytiva). The paper strips were incubated in the culture tubes without touching the culture for 24 h at 37 °C. The paper was colored brown depending on the presence of H_2_S.

### Immunofluorescence (IF) assay

Cells grown on glass coverslips were incubated in bacterial CFS for 24 h before fixation, washed in PBS, and fixed in PBS containing 4% paraformaldehyde for 20 min. Fixed cells were washed with PBS three times, permeabilized with PBS containing 0.1% Triton X-100 (PBT) for 10 min, and blocked with PBS containing 1% bovine serum albumin (BSA) and 0.1% Triton X-100 (BBT) for 1 h. Next, the cells were incubated with a specific primary antibody for 1 h at room temperature, followed by three washes with PBT. Subsequently, cells were incubated with a secondary antibody for 45 min at room temperature. Next, the cells were washed with PBT and incubated with 10 μg/ml 4′,6-diamidino-2-phenylindole (DAPI; Sigma) for 5 min to stain the cell nuclei. Finally, the cells were washed with PBS and mounted onto glass slides. IF analysis was performed using confocal laser microscopy. The following primary antibodies were used: mouse anti-total β-catenin (1:250; BD), Phalloidin-594 (1:1500; Abcam), and Rabbit anti-YAP (1:200; Cell Signaling). Alexa green (1:500; Stratagene) was used as a secondary antibody. The quantification of nuclear β-catenin was established using QuPath software or a self-developed detector that incorporated an image-processing technique and capabilities for measuring RGB units.

### Western blot and co-immunoprecipitation (IP) assay

Cells were washed with PBS and harvested in M2 lysis buffer (100 mM NaCl, 50 mM Tris pH 7.5, 1% Triton X-100, 2 mM EDTA) containing a protease inhibitor cocktail (Sigma-Aldrich). The cell lysates were incubated on ice for 15 min, during which they were homogenized 3 times by vortexing and then centrifuged at 14,000 rpm for 15 min at 4 °C. Protein amount was determined using the Bradford protein assay (Bio-Rad Laboratories Ltd., Hercules, CA, USA) according to the manufacturer's instructions. Equal protein amounts were separated by SDS‒polyacrylamide gel electrophoresis (SDS-PAGE) using 7.5% or 15% gels and then transferred to nitrocellulose membranes. After blocking with 5% low-fat milk in PBS containing 0.001% Tween-20 (PBST), the membranes were incubated overnight at 4 °C with specific primary antibodies (as indicated). Next, the membranes were washed three times for 10 min each in PBST and incubated with the appropriate horseradish peroxidase (HRP)-conjugated secondary antibody (as indicated) for 1 h at room temperature. Subsequently, the membranes were subjected to enhanced chemiluminescence detection analysis. The following antibodies were used for western blot analysis: mouse anti-Snail (1:1000, Cell signaling), rabbit anti-Slug (1:1000, Cell signaling), Rabbit anti-Axin2 (1:1000, Abcam), mouse anti-total-β-catenin (1:1000; BD Transduction Laboratories, Lexington, KY, USA), Rabbit anti-E-cadherin (1:1000, Cell Signaling), Rabbit anti-Yap (1:000, Cell signaling), Rabbit anti P-Yap(S127) (1:000, Cell signaling), Rabbit anti P-Yap(S397) (1:000, Cell signaling), mouse anti-Tubulin (1:10000; Sigma). CC3 (5A1E) Rabbit Monoclonal #9654 (1:1000, Cell Signaling), N-Cadherin (13A9) Mouse Monoclonal Antibody #14215 (1:600, Cell Signaling), Vimentin (D21H3) Rabbit Monoclonal Antibody #5741; (1:1000, Cell Signaling), Fibronectin Antibody (EP5): sc-8422 (1:1000, Santa Cruz), Collagen1clone 5D8-G9 #MAB3391 (1:1000), Integrin a5 (C-9): sc-376199 (1:1000, Santa Cruz), Vinculin Mouse monoclonal, hVIN-1 # V9131 (1:1000, Sigma-Aldrich), Paxillin # 610052 (1:1000, BD Transduction Laboratories™), ZO1 Rabbit Polyclonal Antibody # 61-7300 (1:1000, Thermo). HRP–conjugated secondary antibodies used: anti-Mouse and anti-Rabbit (1:10000; Jackson ImmunoResearch, West Grove, PA, USA). For the co-IP assays, equal amounts of protein were incubated overnight with the indicated primary antibody or serum control in rotation at 4 °C, followed by 2 h of rotation with protein A/G plus agarose beads (Santa Cruz Biotechnology) at 4 °C. Subsequently, the beads were collected by centrifugation, washed three times in M2 lysis buffer containing a protease inhibitor cocktail, and subjected to SDS‒PAGE analysis, followed by detection with specific antibodies (as indicated).

### Luciferase assay

To test β-catenin-mediated transcriptional activation, the cells were grown in a 24-well plate and transfected at 70% confluence with pTOPFLASH/pFOPFLASH and SV40-Rnl plasmids. The total amount of DNA was kept constant by adding the respective empty vectors, as indicated. Forty-eight hours following transfection, the cells were washed with phosphate-buffered saline (PBS) and then lysed using reporter luciferase buffer (Promega) containing a protease inhibitor cocktail (Sigma-Aldrich). Luciferase activity was determined according to the manufacturer's instructions, and the results were normalized to the respective SV40-Rnl values. β-catenin-mediated transcription levels were calculated by determining the relative luciferase activity (pTOPFLASH/pFOPFLASH ratio) in each transfection reaction. All the assays were performed in triplicate, and at least three independent experiments were carried out for each analysis.

### RNA extraction

According to the manufacturer's protocol, RNA was extracted using the Direct-zol RNA MiniPrep Kit (ZYMO RESEARCH). The concentration and purity of the RNA samples were determined, and total RNA was reverse-transcribed using the iScript cDNA Synthesis Kit (Bio-Rad) according to the manufacturer's protocol.

### Quantitative polymerase chain reaction (qPCR)

All the qPCR reactions were performed using the CFX Connect Maestro Real-Time PCR Detection System (Bio-Rad), and the amplifications were carried out using iTaq Universal Supermix (Bio-Rad). The thermal cycling conditions consisted of an initial denaturation step at 95 °C for 10 min, followed by 40 cycles of 95 °C for 15 s and 60 °C for 60 s, with each cycle ending with a plate read. Finally, a melt curve step was performed. The expression level of the tested genes was analyzed using the ΔΔCT technique (normalization to the β-actin expression of each sample, followed by normalization to the control group of the experiment (Wilkins broth)). The primers for the amplification of the specific cDNA sequences were:

**Table:** 

Gene	Primer	Sequence (5′->3′)
Snail	Forward	TCGGAAGCCTAACTACAGCGA
	Reverse	AGATGAGCATTGGCAGCGAG
Slug	Forward	ACTCACACGGGGGAGAAG
	Reverse	TGTGCAGGAGAGACATTCTG
Axin2	Forward	GCAGCTCAGCAAAAAGGGAAAT
	Reverse	TACATCGGGAGCACCGTCTCAT
Fibronectin	Forward	CCAGCAGAGGCATAAGGTTC
	Reverse	CCAGCAGAGGCATAAGGTTC
N-cadherin	Forward	AGCCAACCTTAACTGAGGAGT
	Reverse	GGCAAGTTGATTGGAGGGATG
Twist1	Forward	TTCTCGGTCTGGAGGATGGA
	Reverse	CCACGCCCTGTTTCTTTGAAT
Vimentin	Forward	TCTACGAGGAGGAGATGCGG
	Reverse	GGTCAAGACGTGCCAGAGAC
Zeb1	Forward	GGCATACACCTACTCAACTACGG
	Reverse	TGGGCGGTGTAGAATCAGAGTC
Acly	Forward	ATCGGTTCAAGTATGCTCGG
	Reverse	GACCAAGTTTTCCACGACGTT

### Cell viability assay

HCT116 cells (4.5 × 10^7^ cells) were seeded in a 6-well plate. The next day, the cells were incubated with several concentrations of *Pg*-CFS or Wilkins media for 24 h. The cells were harvested and subjected to WB using the Cleaved Caspase3 antibody (Cell Signaling). Band quantification was established using ImageJ software. Alternatively, Alamar Blue reagent (Invitrogen) was added to the cells according to the manufacturer's protocol. After incubation for 2 h at 37 °C, 100 µL from each well was transferred to a fresh 96-well plate for fluorescent detection using an Infinite F200 plate reader (TECAN). In both methods, the results were normalized to those of the Wilkins control group.

### Adherent cells assessment assay

HCT116 cells (4.5 × 10^7^ cells) were seeded in a 6-well plate. The next day, the cells were incubated with several concentrations of *P. gingivalis*-CFS or Wilkins media for 24 h. Then, all the media was aspirated (with the suspended cells) and replaced with fresh media containing Alamar Blue reagent (Invitrogen) according to the manufacturer's protocol. After incubation for 2 h at 37 °C, 100 µL from each well was transferred to a fresh 96-well plate for fluorescent detection using an Infinite F200 plate reader (TECAN). The results were normalized to the Wilkins control group.

### Mass-spectrometry analysis

*Pg-*CFS was sent for mass spectrometry analysis (Technion) using Discoverer 1.4 identification with the Sequest (Thermo) search engine. The samples were digested by trypsin, analyzed by LC–MS/MS on Q-Exactive (Thermo), and identified by Discoverer software against *P. gingivalis* unspecific databases.

### Incucyte analysis

HCT116 cells (3.5 × 10^6^ cells) were seeded in a 96-well plate. The next day, a scratch wound was created using a 96-well wound maker (ESSEN Bioscience Inc., Ann Arbor, MI). Subsequently, the cells were washed twice with PBS, and *P. gingivalis*-CFS was added as indicated. The wound healing was quantified using IncuCyte. Blinded analysis of cell motility parameters was performed by tracking cellular movements during the scratch assay. Snapshots were taken every 2 h using IncuCyte. Thereafter, the images were collected, and single-cell analysis of motility parameters was performed using IMARIS with the Imaris surface mode, as previously described.^119^ At least 100 cells were analyzed.

### Statistical analysis

Statistical analyses were performed with GraphPad Prism version 9 (GraphPad Software). The experimental data are expressed as mean ± standard deviation (SD), and specific statistical tests are detailed in the figure legends. A two-tailed Student's *t*-test was performed to compare normally distributed continuous variables, and a two-tailed Mann‒Whitney U test was used for non-normal distributions. When *t*-tests were performed multiple times, multiple testing correction was carried out. For experiments with more than two groups, the comparison was performed with one- or 2-way ANOVA with Holm–Šídák's or Dunn's post-test. The Holm–Šídák's post-hoc test was used to assess the significance of predefined comparisons between groups at specific time points.

## Supplementary Material

Supplementary material Supplementary_Tables.docx

Supplementary material Supplementry_FigsCleanVersion.docx

Supplemental Material Supplementary_caption.docx

## Data Availability

Published on FigShare: The data that support the findings of this study are openly available at: https://figshare.com/articles/dataset/Figures/29435360?file=55763822.
