# Supplementary material for: Porphyromonas gingivalis secreted factors drive epithelial–mesenchymal transition (EMT) through gingipains and an H2S-mediated bacterial defense system
Source: Gut Microbes. 2026 Mar 24;18(1):2647532. doi: 10.1080/19490976.2026.2647532 (PMC13014561; doi:10.1080/19490976.2026.2647532)

Supplementary Table 1:

| **Condition** | **Motility Type on Continuum** | **Reasoning Based on Obtained Data** |
| --- | --- | --- |
| Control (0%) | Epithelial | High adherence (~100%), moderate migration velocity, moderate ellipticity, high E-cadherin (epithelial junctions intact), low nuclear β-catenin and YAP activation, baseline EMT markers (Slug, Snail, Vimentin, Fibronectin). |
| 20% Pg-CFS | Partial EMT (pEMT) | Moderate reduction in adhesion (~80%), increased velocity, higher displacement and MSD, slightly rounder cells, decreased E-cadherin, intermediate nuclear β-catenin activation, moderate induction of EMT transcription factors (Slug, Snail), and significant increase in Vimentin and Fibronectin. |
| 40% Pg-CFS | EMT with transition towards Ameboid | Significantly reduced adherence (~20%), further increased migration velocity, intermediate displacement2 and MSD (suggesting high local mobility but less linear displacement), sphericity returns to baseline despite severe adhesion loss, strongly reduced E-cadherin, very high nuclear β-catenin and YAP (high Wnt signaling activation), dramatic upregulation of EMT markers (Slug, Snail), significant upregulation of Vimentin and Fibronectin, indicating strong mesenchymal transition and high potential for individual cell motility consistent with ameboid features. |

Based on the comprehensive morphokinetic and molecular data, the detailed classification of HCT116 cells (Control, 20% *Pg*-CFS, and 40% *Pg*-CFS) placed on the motility continuum from epithelial to ameboid motility.

**Supplementary Table 2.** Mass spectrometric analysis of polypeptides expressed in the CFS obtained from *P. gingivalis*


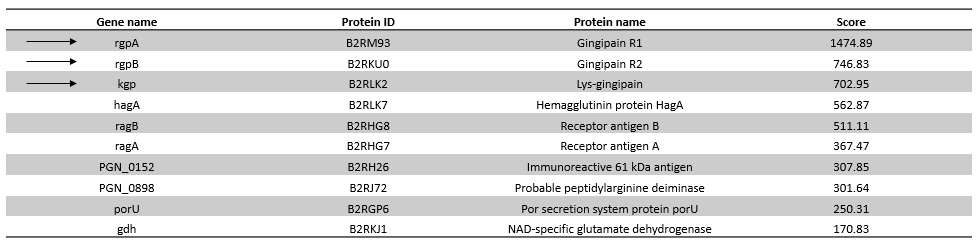

Supplement: Supplementary material — Supplementary_Tables.docx [file KGMI_A_2647532_SM3923.docx]
