# Supplementary material for: Porphyromonas gingivalis secreted factors drive epithelial–mesenchymal transition (EMT) through gingipains and an H2S-mediated bacterial defense system
Source: Gut Microbes. 2026 Mar 24;18(1):2647532. doi: 10.1080/19490976.2026.2647532 (PMC13014561; doi:10.1080/19490976.2026.2647532)
Supplement: Supplementary material — Supplementry_FigsCleanVersion.docx [file KGMI_A_2647532_SM3922.docx]

**Fig. S1:** The CFS of *P. gingivalis* does not affect Wnt signaling activity, Axin-2 or Slug in non-CRC HEK293 cells.

## A

5

ns

4

Wnt signaling activity

3

2

1

0

Cb Ec Pg Cb Ec Pg

B 20% CFS

Cb Ec Pg

2.0

slug

Axin2 Slug Tubulin

Control 4h 24h

4h 24h

4h 24h

KDa 94

35


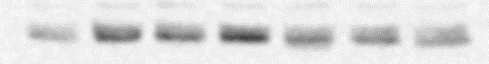

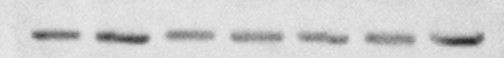

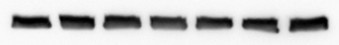


55

1.5

1.0

Relative expression (AU)

0.5

0.0

Cb Ec Pg Cb Ec Pg

0.0131

0.0002

0.0084

<0.0001

40% CFS

2.0

slug

## C

Axin2

Slug Tubulin

Control

Cb Ec 4h 24h 4h 24h

Pg 4h 24h

KDa 94


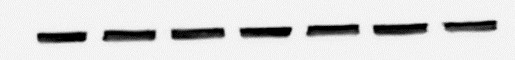

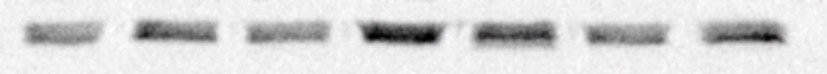

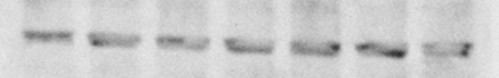


35

55

1.5

1.0

Relative expression (AU)

0.5

0.0

Cb Ec Pg Cb Ec Pg

**Fig. S2:** Cell viability following *Pg* CFS treatment (Alamar Blue)

ns

1.2

0.8

Relative viability

0.4

0.0

20%

40%

60%

80%

100%

**Fig. S3:** MMPs are not involved in the effect of the *Pg*-CFS on EMT

B ns

A

**8**

Wilkins *Pg*


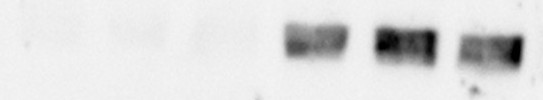

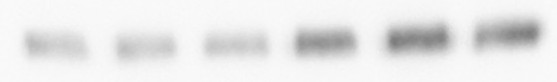

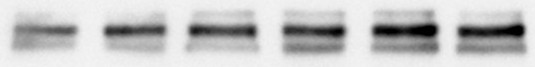


Luciferase activity

Slug **6**

Snail **4**

Actin

**2**

GM6001 (µM)

0 10 20 0 10 20

# 20% CFS

**0** - DMSO 4h 24h

GM6001

**Fig. S4:** *Pg* CFS does not affect Axin2 protein levels

20% CFS 40% CFS

4 1.5

ns

ns

Relative WB levels (AU)

Relative Wb levels (AU)

3

1.0

2

0.5

1

4h 24h

0

Cb Ec Pg Cb Ec Pg

0.0

Cb Ec Pg Cb Ec Pg

**Fig. S5:** Snail is upregulated by *P. gingivalis*-CFS

20% CFS

## A

4

<0.0001

<0.0001

0.0047

3

Reltive RNA levels

2

1

0

Cb Ec Pg Cb Ec Pg

## B

20% CFS

40% CFS

8

<0.0001

<0.0001

0.0028

0.0028

6

Reltive RNA levels

4

2

0

Cb Ec Pg Cb Ec Pg

4

0.0002

0.0019

<0.0001 0.0004

Snail

Cb Control 4h 24h

Ec

4h 24h

Pg

4h 24h

3

KDa

Relative expression (AU)

2


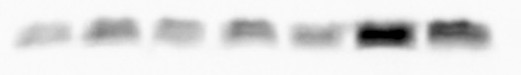


35

1


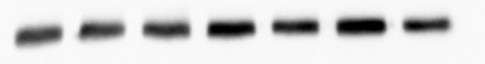


Tubulin 55

0

Cb Ec Pg Cb Ec Pg

0.0487

0.0204

C 40% CFS

Cb Ec Pg

5 0.0243

4h 24h

Snail Tubulin

Control 4h 24h

4h 24h

4h 24h

KDa 4

3


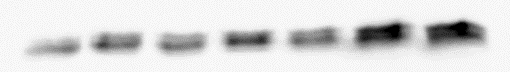


Relative expression (AU)

35

2


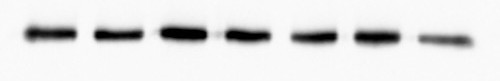


55 1

0

Cb Ec Pg Cb Ec Pg

**Fig. S6** Optimization of Leupeptin concentration.

|  | Control 40% | CFS | KDa |
| --- | --- | --- | --- |
| Slug | 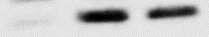 | 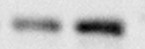 | 35 |
| Snail | 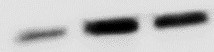 | 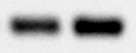 | 35 |
| Tubulin | 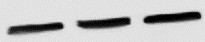 | 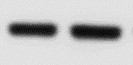 | 55 |

Leupeptin (mM)

|  |  |
| --- | --- |
|  |  |
|  |  |

0 0 0.5 1 2

**Fig. S7:** the effect of 20% *P. gingivalis*-CFS under H_2_S inhibition

1. NL1 (mM)


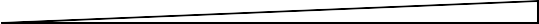


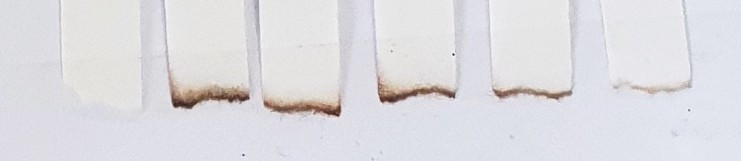
Control 0

0.5 0.75

1 1.5

# 20% CFS


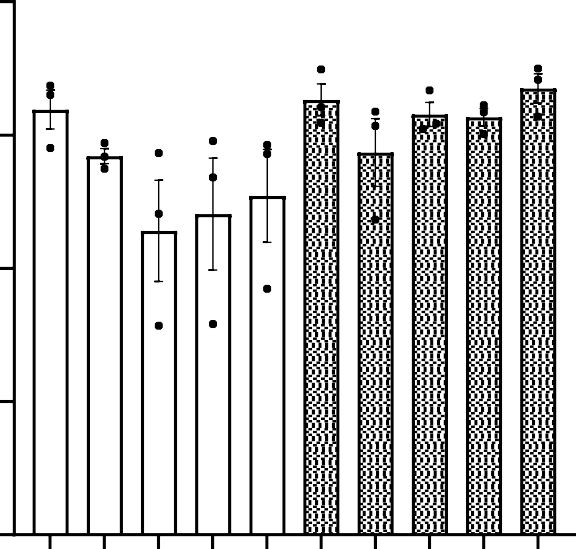
80

Precentage of adherent cells (%)


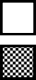
60

40

20

Anaerobic Oxygen stress

0

Pg-CFS - + + + + - + + + +


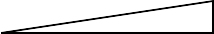
NL1
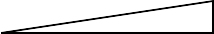


Fig. S8: The effects of the gingipains and H_2_S do not synergizes

## Control NL1 (0.5mM)


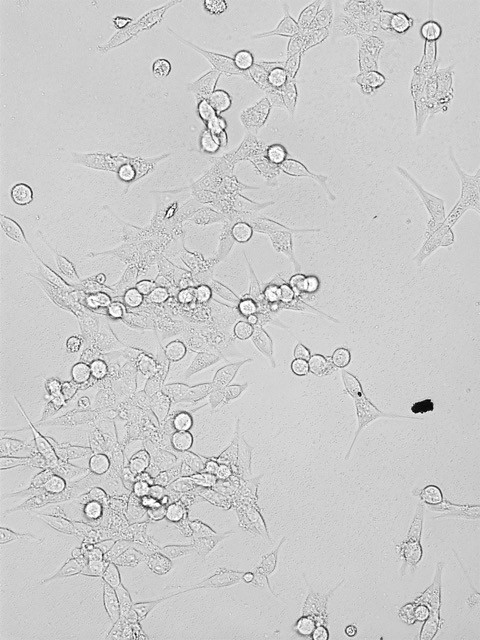

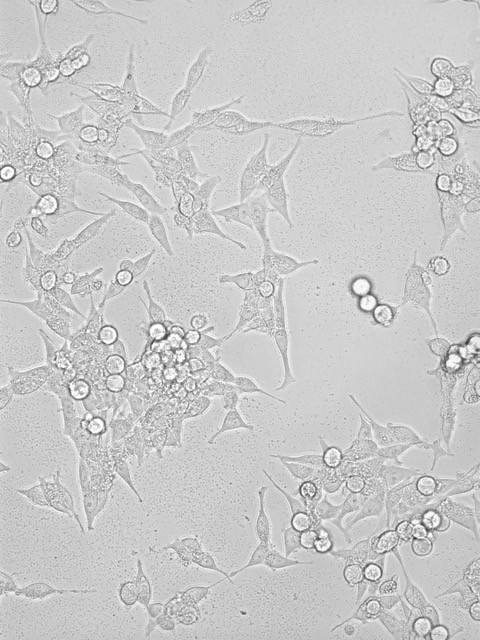

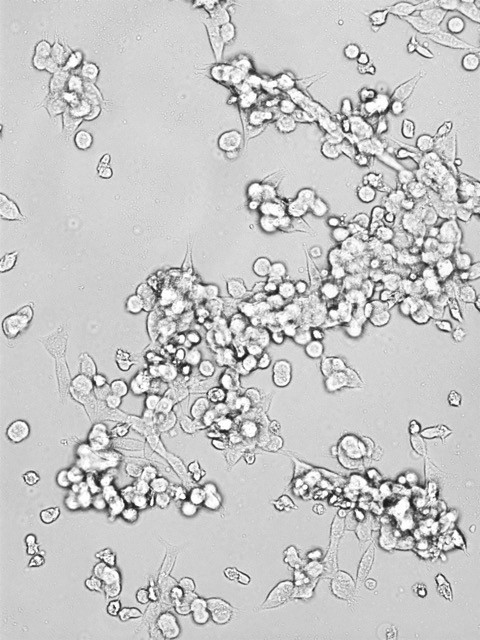

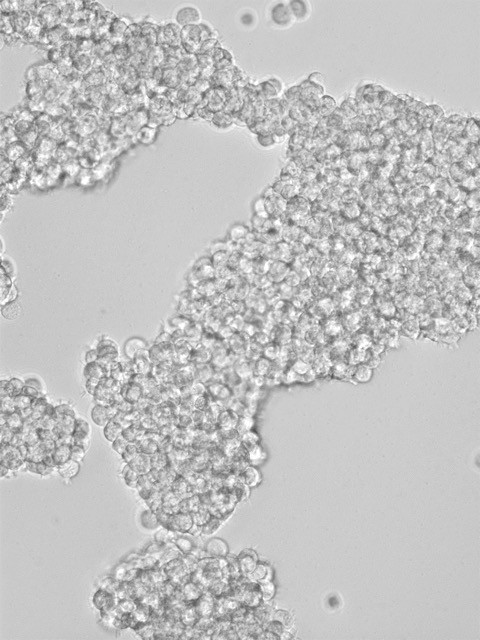

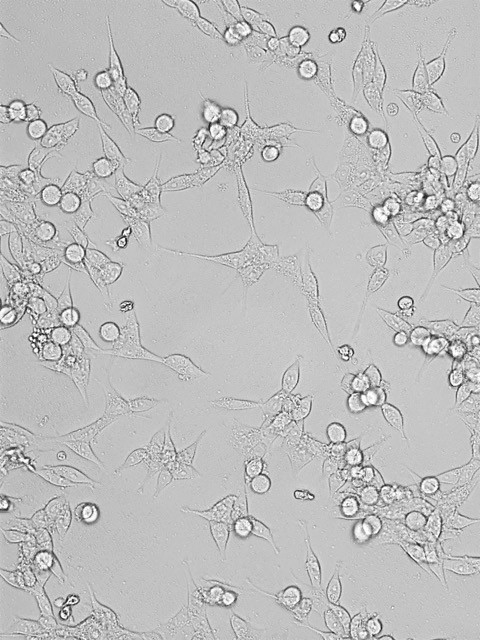

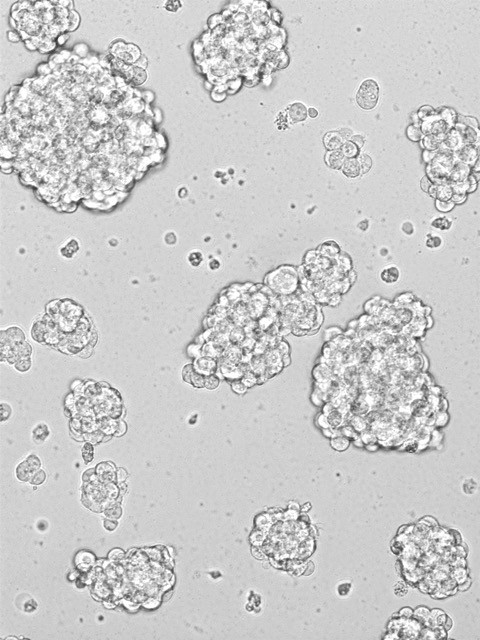

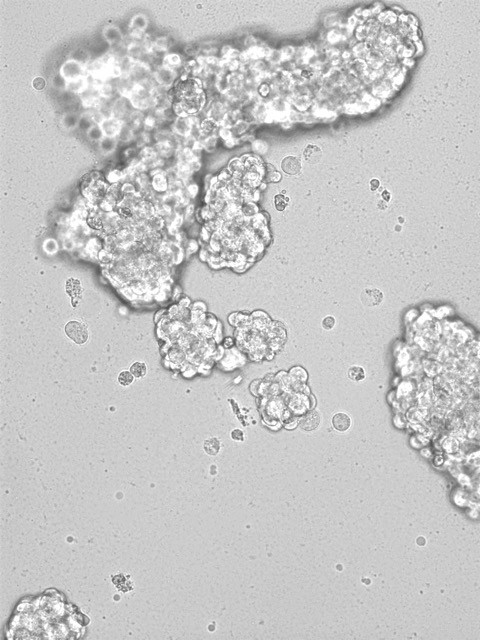

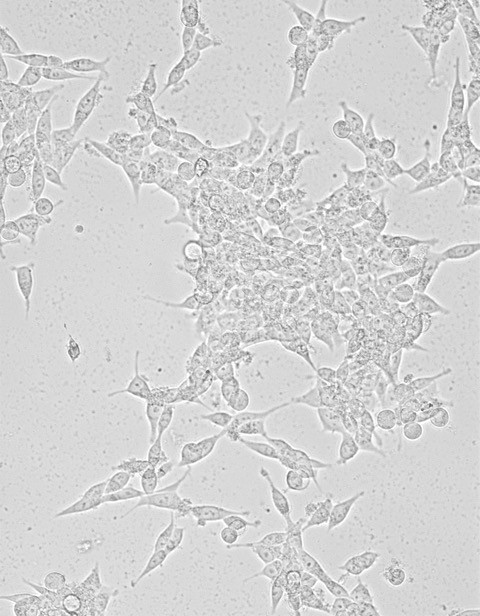


*Pg*-WT

*Pg*-Kgp-

*Pg*-RgpB-

*Pg*-RgpA-

Fig. S9: The effect of the Pg-CFS on Wnt signaling activation is not affected by H_2_S depletion

**10**

ns

**8**

Relative Wnt activity

**6**

**4**

**2**

**0**

**Control**

**Pg-20%**

**NL1**

**Fig. S10**: Bacteria viability by Propidium Iodide staining.


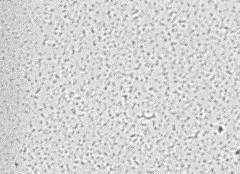

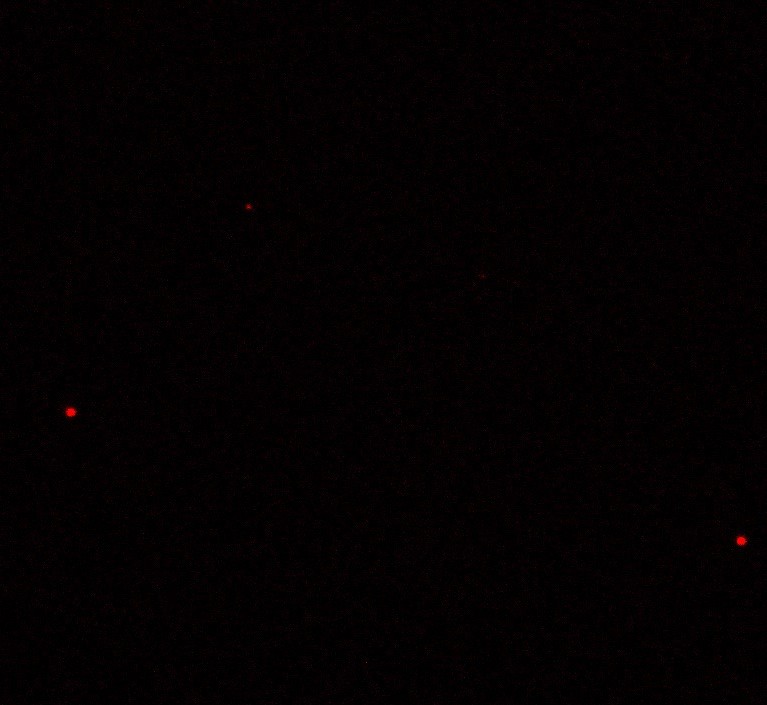
*Pg* at OD=1.8 PI staining


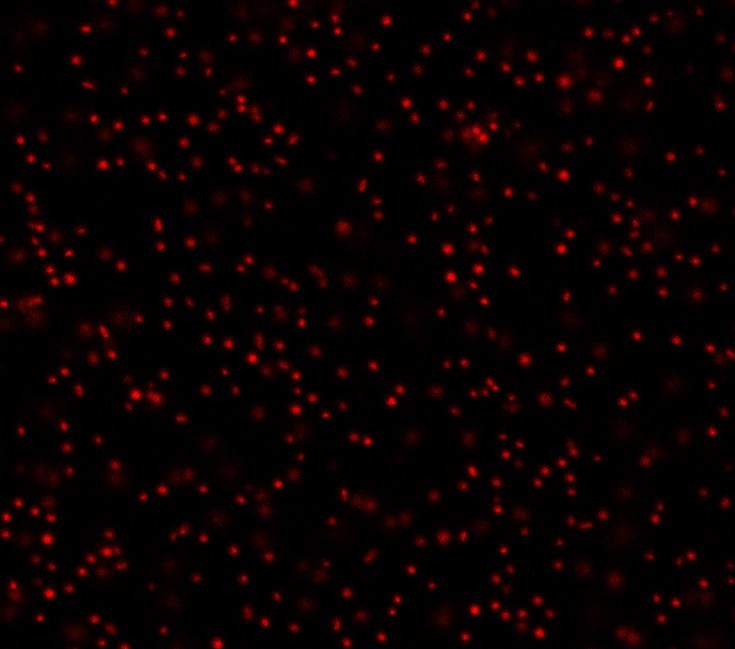


EtOH treatment
