# Supplementary material for: Porphyromonas gingivalis secreted factors drive epithelial–mesenchymal transition (EMT) through gingipains and an H2S-mediated bacterial defense system
Source: Gut Microbes. 2026 Mar 24;18(1):2647532. doi: 10.1080/19490976.2026.2647532 (PMC13014561; doi:10.1080/19490976.2026.2647532)
Supplement: Supplemental Material — Supplementary_caption.docx [file KGMI_A_2647532_SM2391.docx]

**Fig. S1. The CFS of *P. gingivalis* does not affect Wnt signaling activity, Axin2, or Slug in non-CRC HEK293 cells.** (**A**) HEK293T cells were treated with 20% CFS from *C. butyricum*, *E. coli,* and *P. gingivalis* bacteria for six hours or 24 hours. The cells were harvested and subjected to the TOPFLASH/FOPFLASH Luciferase assay. The results were normalized to the Wilkins broth control. (**B-C**) HEK293T cells were incubated with 20% (**B**) or 40% (**C**) CFS from *C. butyricum, E. coli,* and *P. gingivalis* bacteria for four h or 24h. The cells were harvested and subjected to WB analysis using antibodies against Axin2 and Slug. Tubulin was used as a loading control. Band quantification was calculated using ImageJ software. Two-way ANOVA with Holm-Šídák's multiple comparisons test was employed. P-values are indicated in the graph. Pg = P. gingivalis, Ec = E. coli, Cb = C. butyricum.

**Fig. S2. Cell viability following *Pg* CFS treatment (Alamar Blue).** HCT116 cells were treated with increasing concentrations of *Pg*-CFS (0-100%) for 24h. Cellular adherence was determined by Alamar Blue analysis as described in the method section. The results were normalized to the Wilkins broth control.

**Fig. S3. MMPs are not involved in the effect of the on EMT.** (**A**) HCT116 cells were treated with 20% *Pg*-CFS supplemented with 10 µM or 20 µM GM6001 for 24 hours; DMSO was used as a control. The cells were harvested and subjected to WB analysis using the indicated antibodies. Actin was used as a loading control, and the results were normalized to the Wilkins broth control. (**B**) HCT116 cells were transfected with the TOPFLASH/FOPFLASH system vectors following incubation with 20% *Pg*-CFS and 25µM GM6001 for 4h or 1µM GM6001 for 24h (DMSO was used as a control). The cells were harvested and subjected to a Luciferase assay. The results were normalized to the Wilkins broth control. Two-way ANOVA with Holm-Šídák's multiple comparisons test was employed.

**Fig. S4. *Pg*-CFS does not affect Axin2 protein levels.** HCT116 cells were treated with 20% or 40% CFS from C. butyricum, *E. coli,* and P*. gingivalis* bacteria for 4h or 24 hours. The cells were harvested and subjected to WB analysis using the indicated antibodies (presented in Fig. 3B-C). Band quantification was established and normalized to the Wilkins broth control using ImageJ software. Two-way ANOVA with Holm-Šídák's multiple comparisons test was employed.

**Fig. S5. Snail is upregulated by *Pg*-CFS.** (**A**) HCT116 cells were treated with 20% (left) or 40% (right) CFS from *C. butyricum*, *E. coli,* and *P. gingivalis* bacteria for four h or 24h, and Snail gene expression was determined by qPCR analysis. Actin was used as a control. (**B-C**) HCT116 cells were treated with 20% (**B**) or 40% (**C**) CFS from *C. butyricum*, *E. coli,* and *P. gingivalis* bacteria for 4h or 24h. The cells were harvested and subjected to WB analysis using the indicated antibodies. Tubulin was used as a loading control. Band quantification was calculated using ImageJ software. The results were normalized to the Wilkins broth control. Two-way ANOVA with Holm-Šídák's multiple comparisons tests were employed; P-values are indicated in the graphs. Pg = *P. gingivalis*, Ec = *E. coli*, Cb = *C. butyricum.*

**Fig. S6. Optimization of Leupeptin concentration.** HCT116 cells were treated with control and 40% *Pg*-CFS for 24h, supplemented with leupeptin (0.5-2mM) as described in the methods section. The cells were harvested and subjected to WB analysis using the indicated antibodies.

**Fig. S7. The effect of 20% *Pg* CFS under H_2_S inhibition. (A)** *P. gingivalis* bacteria were cultured for 24h with increasing concentrations of the NL1 inhibitor. Lead Acetate: Whatman indicator papers indicate the presence of gaseous H_2_S. (**B**) *P. gingivalis* bacteria were cultured anaerobically or under oxygen stress for 72h with similar NL1 concentrations (as in A). The bacterial CFSs were used to treat HCT116 cells for 24h. Cellular adherence was determined by Alamar Blue analysis. The results were normalized to the Wilkins control. Two-way ANOVA with Holm-Šídák's multiple comparisons test was employed.

**Fig. S8. The effects of the gingipains and H_2_S do not synergize.** CFS from wild-type *P. gingivalis* and the *kgp^-^*, *rgpB*^-,^ and *rgpA*^-^ mutant strains cultured in oxygen stress for 72h and treated with 0.5mM NL1, was used to treat HCT116 cells for 24h. Cellular detachment was visualized by light microscopy.

**Fig. S9. The effect of the *Pg*-CFS on Wnt signaling activation is not affected by H_2_S depletion.** HCT116 cells were transfected with the TOPFLASH/FOPFLASH system vectors following 24h incubation with *Pg-CFS* or CFS obtained following treatment with 1mM NL1. The cells were harvested and subjected to a Luciferase assay. The results were normalized to the Wilkins broth control. Kruskal-Wallis ANOVA with Dunn's multiple comparisons test was employed.

**Fig. S10.** **Bacteria viability by Propidium Iodide staining.** The viability of the treated bacteria was determined by Propidium Iodide staining (PI). EtOH treatment was used to induce membrane damage and served as a control for dead cells.

**Supplementary Table 1:** Classification of HCT116 cells (Control, 20% *Pg*-CFS, and 40% *Pg*-CFS) placed on the motility continuum from epithelial to ameboid motility.

**Supplementary Table 2:** **Mass spectrometric analysis of polypeptides expressed in the *Pg*-CFS.** The CFS collected from *P. gingivalis* bacteria was sent for mass spectrometry analysis. The proteins detected were organized by frequency score, from highest to lowest.
